# 

**Published:** 1889-02

**Authors:** 


					﻿1889,
OLD SERIES
Vol. xxv
NEW SERIES
Vol. V.—No. 121
& 1855 O
Mt wk
Hedigal^w^K
(atOURNABf
hx etj£S^ai^8BSS4^ore 5
^TXvV^Sttvr/Wt&u.^-db-S.

W.F.WESTMORELAND.M D.
H.V.M.MILLER.M.D.LL.D^
■Published By james p.harrison&cqS
a? ____________ «Stlanta,ga. Js
SUBSCRIPTION c $2.00 A YEAR.
				

